# Novel assembly of the SF370 strain of the important human pathogen *Streptococcus pyogenes* serotype M1

**DOI:** 10.1128/mra.01197-24

**Published:** 2024-12-12

**Authors:** Thomas Fabian Wulff, Rina Ahmed-Begrich, Karin Hahnke, Michael Jahn, Emmanuelle Charpentier

**Affiliations:** 1 Max Planck Unit for the Science of Pathogens, Berlin, Germany; 2 Institute for Biology, Humboldt University Berlin, Berlin, Germany; University of Maryland School of Medicine, Baltimore, Maryland, USA

**Keywords:** *Streptococcus pyogenes*, hybrid assembly

## Abstract

The important human pathogen *Streptococcus pyogenes* causes a wide range of diseases. Strain SF370 was the first fully sequenced strain of *S. pyogenes*, providing essential insights into the molecular mechanisms of disease. We present an improved genome assembly of strain SF370 from ATCC, which corrects long-standing errors in its genome.

## ANNOUNCEMENT

*Streptococcus pyogenes* is a major human pathogen, causing over 700 million superficial infections and 600,000 severe invasive infections annually (1). Strain SF370 is representative of serotype M1, the most prevalent serotype in high-income countries (2), and was the first fully sequenced *S. pyogenes* isolate (3, 4). Our laboratory has previously encountered difficulties in mapping sequencing data to the tRNA-encoding regions of the SF370 genome using the current reference sequence (NC_002737.2) (4, 5). We have, therefore, re-sequenced strain SF370 using long- and short-read sequencing and present here an improved genome assembly.

Strain SF370 was obtained from ATCC (ATCC 700294) in 2013 and stored at −80°C in 20% glycerol without passaging. Bacteria were streaked on Trypticase Soy Agar plates with 3% defibrinated sheep blood and then grown in Todd Hewitt Broth with 0.2% yeast extract at 37°C and 5% CO_2_ overnight. The pre-culture was diluted 1/100 in fresh medium and grown for 7 h. Cells were pelleted, washed, and stored at −20°C.

For nanopore sequencing, genomic DNA was extracted using the Monarch HMW DNA Extraction Kit for Tissue (NEB). Libraries were prepared using the Native Barcoding Kit (SQK-NBD114.24) with 1 µg of needle sheared DNA (G27, 15 passages, no size selection) and sequenced using an R10.4.1 flow cell (FLO-MIN114) on a MinION Mk1c (ONT). Basecalling and demultiplexing were performed using Dorado (v0.5.3) with the super accurate model (v4.3.0) (447k reads, N_50_ of 18.9 kb; 1,200× coverage). For Illumina sequencing, genomic DNA was extracted using the NucleoSpin Microbial DNA Mini Kit (Macherey-Nagel). Libraries were prepared using the Illumina DNA Prep Kit with 250 ng of DNA and sequenced on an Illumina MiSeq (paired-end mode, 300 bp; 33.7 M reads, 4,850× coverage). Read quality was assessed using NanoPlot (v1.40.0) (6) and FastQC (v0.11.9) (7). Hybrid assembly was performed with Hybracter (v0.7.3) using default parameters (8), relying on the Flye assembler for contig circularization and dnaapler for *dnaA*-based reorientation (9, 10).

**Fig 1:**
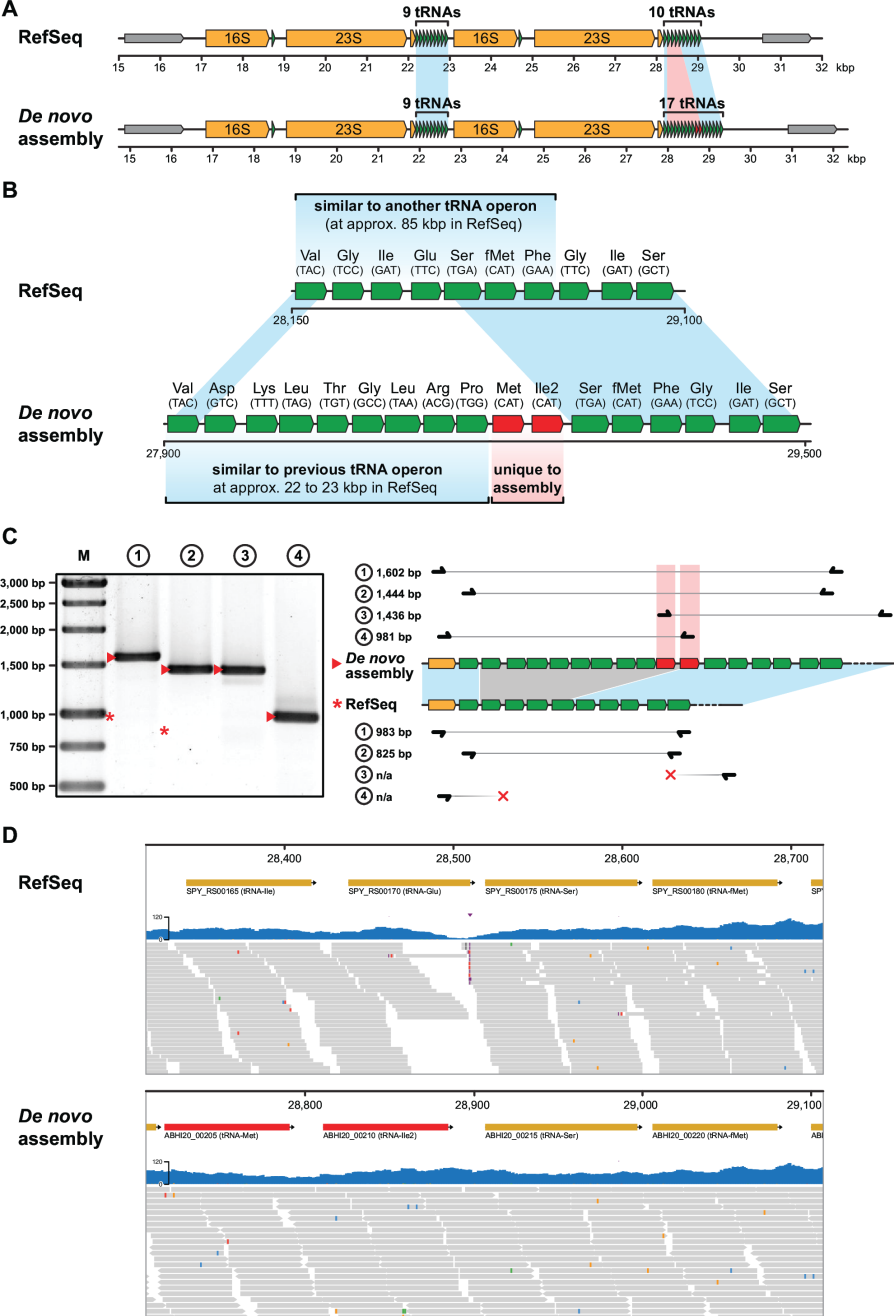
Correction of the tRNA operon in *S. pyogenes* SF370. (**A**) The tandem rRNA-tRNA operon in *S. pyogenes* SF370 as organized in the current reference sequence (NC_002737.2; upper part) and in the new assembly described here (lower part). rRNA genes are depicted in orange and tRNA genes in green. Similar regions within the tRNA operons are highlighted in light blue, differing regions in light red. (**B**) Detailed view on the tRNA operon differing between the current reference sequence and our assembly. For both RefSeq and assembly, regions of the tRNA operon that are similar to other tRNA operons in the genome are highlighted in blue with a reference to the respective region. (**C**) Validation of the tRNA operon structure by PCR. Primers amplifying the region of interest within the tRNA operon were designed as shown on the right (Table 1), and the resulting PCR amplicons were purified and analyzed by 1% agarose gel electrophoresis (left panel; GeneRuler 1 kb as marker “M”). Amplicon sizes expected for the assembly are marked by a triangle and for the RefSeq with a star. PCR amplicons support the updated structure of the tRNA operon. (**D**) Exemplary alignment view on the region of interest within the updated tRNA operon. Illumina sequencing data used for the current RefSeq version of the *S. pyogenes* genome (4) was aligned using BWA-MEM (v0.7.18) (15) and visualized using SpyViewer. It should be noted that the sequencing reads do not align continuously to the current reference sequence but do align well to our assembly.

The assembled genome consisted of a single contig of 1,853,025 bp with a GC content of 38.51%. The NCBI Prokaryotic Genome Assembly Pipeline (v6.7) (11) annotated 1,777 coding sequences, 18 rRNAs, and 67 tRNAs. The assembly and related sequencing data can be browsed interactively using SpyViewer (https://spyviewer.mpusp.mpg.de). Comparison of the new assembly with the current reference sequence revealed several differences in the coding sequences (mostly transposase genes) and the six rRNA-tRNA operons (see the variant track in SpyViewer). In particular, we identified a major assembly artifact in a tandem rRNA-tRNA operon (Fig. 1A). The reference sequence erroneously included 10 tRNA genes at the end of the operon (partially reminiscent of another tRNA operon), whereas our assembly confirms the presence of 17 tRNA genes. Importantly, our assembly harbors two additional tRNAs (tRNA^Ile2^ and elongator tRNA^Met^) that are absent from the reference sequence (Fig. 1B) (12–14). We validated the structure of the tRNA operon using PCR (Fig. 1C; Table 1) and further showed that the sequencing data used to generate the current reference sequence are consistent with our assembly (Fig. 1D). In conclusion, re-sequencing of *S. pyogenes* SF370 corrects long-standing assembly errors and provides a new, highly reliable genome sequence.

**TABLE 1:** Primer sequences used to verify the tRNA operon structure^*a*^

	Primer code	Primer sequence	F/R	*T*_annealing_	*t*_elongation_	Expected amplicon length according to…	
						RefSeq	Assembly
1	OLEC15166	GCCGAACACAGCAGTTAAGC	F	65.0°C	50 s	983 bp	1,602 bp
	OLEC15167	CGCCAGTTACCCGACCTAAC	R				
2	OLEC15168	TTACAAGCAGAGGGTCAGCG	F	65.0°C	50 s	825 bp	1,444 bp
	OLEC15169	CCGTCCTCTTCAGCCTCTTG	R				
3	OLEC15170	GCTAGAGCGTCCGGTTCATA	F	65.0°C	50 s	—	1,436 bp
	OLEC15171	TGTAAATGCTGGGGACGCAA	R				
4	OLEC15172	TGATGTAGTTGGGGGTTGCC	F	65.4°C	30 s	—	981 bp
	OLEC15173	AGCCGAGTGCTCTAACCAGT	R				

^*a*^ Target sequences were amplified from genomic DNA in 50 µL reactions of 1× Phusion HF Buffer with 0.2 mM dNTPs, 0.5 µM of each primer, and 1 U of Phusion High-Fidelity DNA Polymerase according to the following cycling parameters: 30 s at 98°C; 30 cycles consisting each of 10 s at 98°C, 30 s at the respective annealing temperature (*T*_annealing_), and an amplicon-specific elongation time (*t*_elongation_) at 72°C; and 5 min at 72°C. Amplicon numbers correspond to those shown in Figure 1. The expected amplicon lengths based on the current reference sequence NC_002737.2 and the new *de novo* assembly presented in this manuscript (CP160045) are shown. The “—" sign indicates that no amplification has taken place due to the absence of primer-binding sites. F/R corresponds to forward or reverse primer, respectively.

## Data Availability

Raw read sequences have been deposited under accession numbers ERR12685545 (ONT) and ERR12685632 (Illumina) as part of BioProject number PRJNA1175669. The genome sequence is available from GenBank under accession number CP160045. The genome assembly and related sequencing data can also be accessed and interactively browsed using SpyViewer (https://spyviewer.mpusp.mpg.de). The Illumina sequencing data used in Fig. 1D are from accession number PRJNA236767 (SRX455423; SF370 referred to as MGAS26474).

## References

[1] Carapetis JR, Steer AC, Mulholland EK, Weber M. 2005. The global burden of group A streptococcal diseases. Lancet Infect Dis 5:685–694. doi:10.1016/S1473-3099(05)70267-X 16253886

[2] Steer AC, Law I, Matatolu L, Beall BW, Carapetis JR. 2009. Global emm type distribution of group A streptococci: systematic review and implications for vaccine development. Lancet Infect Dis 9:611–616. doi:10.1016/S1473-3099(09)70178-1 19778763

[3] Ferretti JJ, McShan WM, Ajdic D, Savic DJ, Savic G, Lyon K, Primeaux C, Sezate S, Suvorov AN, Kenton S, Lai HS, Lin SP, Qian Y, Jia HG, Najar FZ, Ren Q, Zhu H, Song L, White J, Yuan X, Clifton SW, Roe BA, McLaughlin R. 2001. Complete genome sequence of an M1 strain of Streptococcus pyogenes. Proc Natl Acad Sci U S A 98:4658–4663. doi:10.1073/pnas.071559398 11296296 PMC31890

[4] Nasser W, Beres SB, Olsen RJ, Dean MA, Rice KA, Long SW, Kristinsson KG, Gottfredsson M, Vuopio J, Raisanen K, Caugant DA, Steinbakk M, Low DE, McGeer A, Darenberg J, Henriques-Normark B, Van Beneden CA, Hoffmann S, Musser JM. 2014. Evolutionary pathway to increased virulence and epidemic group A Streptococcus disease derived from 3,615 genome sequences. Proc Natl Acad Sci U S A 111:E1768–E1776. doi:10.1073/pnas.1403138111 24733896 PMC4035937

[5] Lautenschläger N, Schmidt K, Schiffer C, Wulff TF, Hahnke K, Finstermeier K, Mansour M, Elsholz AKW, Charpentier E. 2024. Expanding the genetic toolbox for the obligate human pathogen Streptococcus pyogenes. Front Bioeng Biotechnol 12:1395659. doi:10.3389/fbioe.2024.1395659 38911550 PMC11190166

[6] De Coster W, Rademakers R. 2023. NanoPack2: population-scale evaluation of long-read sequencing data. Bioinformatics 39:btad311. doi:10.1093/bioinformatics/btad311 37171891 PMC10196664

[7] Andrews S. 2010. FastQC: a quality control tool for high throughput sequence data

[8] Bouras G, Houtak G, Wick RR, Mallawaarachchi V, Roach MJ, Papudeshi B, Judd LM, Sheppard AE, Edwards RA, Vreugde S. 2024. Hybracter: enabling scalable, automated, complete and accurate bacterial genome assemblies. Microb Genom 10:001244. doi:10.1099/mgen.0.001244 38717808 PMC11165638

[9] Kolmogorov M, Yuan J, Lin Y, Pevzner PA. 2019. Assembly of long, error-prone reads using repeat graphs. Nat Biotechnol 37:540–546. doi:10.1038/s41587-019-0072-8 30936562

[10] Bouras G, Grigson SR, Papudeshi B, Mallawaarachchi V, Roach MJ. 2024. Dnaapler: a tool to reorient circular microbial genomes. J Open Source Software 9:5968. doi:10.21105/joss.05968

[11] Tatusova T, DiCuccio M, Badretdin A, Chetvernin V, Nawrocki EP, Zaslavsky L, Lomsadze A, Pruitt KD, Borodovsky M, Ostell J. 2016. NCBI prokaryotic genome annotation pipeline. Nucleic Acids Res 44:6614–6624. doi:10.1093/nar/gkw569 27342282 PMC5001611

[12] Lee CP, Seong BL, RajBhandary UL. 1991. Structural and sequence elements important for recognition of Escherichia coli formylmethionine tRNA by methionyl-tRNA transformylase are clustered in the acceptor stem. J Biol Chem 266:18012–18017. doi:10.1016/S0021-9258(18)55230-3 1917939

[13] Suzuki T, Miyauchi K. 2010. Discovery and characterization of tRNA^Ile^ lysidine synthetase (TilS). FEBS Lett 584:272–277. doi:10.1016/j.febslet.2009.11.085 19944692

[14] Silva FJ, Belda E, Talens SE. 2006. Differential annotation of tRNA genes with anticodon CAT in bacterial genomes. Nucleic Acids Res 34:6015–6022. doi:10.1093/nar/gkl739 17071718 PMC1635315

[15] Li H. 2013. Aligning sequence reads, clone sequences and assembly contigs with BWA-MEM. arXiv. doi:10.48550/arXiv.1303.3997

